# The effects of obesity on 1-year functional outcomes after arthroscopic rotator cuff tear repair

**DOI:** 10.1016/j.jseint.2022.04.004

**Published:** 2022-05-05

**Authors:** Neil Gambhir, Dhruv Shankar, Matthew Alben, Young Kwon, Andrew Rokito, Mandeep S. Virk

**Affiliations:** Division of Shoulder and Elbow Surgery, Department of Orthopedic Surgery, NYU Langone Orthopedic Hospital -NYU Langone Health, New York, NY, USA

**Keywords:** Arthroscopic rotator cuff repair, Functional outcomes, Obesity, Shoulder pain, Shoulder function, Rotator cuff

## Abstract

**Background:**

The purpose of our study was to examine the impact that an increased body mass index (BMI) has on arthroscopic rotator cuff repair (aRCR) outcomes.

**Methods:**

We identified a sample of 313 patients who underwent aRCR at our institution from 2017 to 2020. Patients were classified into cohorts by BMI: normal BMI (<25), overweight (25-30), and obese (≥30). Patient-Reported Outcomes Measurement Information System (PROMIS) scores (Pain Interference, Pain Intensity, and Upper Extremity) and Clinical Global Impressions scale rating of pain and functional improvement after surgery were obtained at 1 year postoperatively. The significance of the BMI category as a predictor for outcomes was evaluated using multiple linear and multivariable logistic regressions. Receiver operating characteristic curve analysis with Youden’s J-statistic was used to determine optimal BMI cutoff for predicting likelihood of achieving minimum clinically important difference (MCID) and substantial clinical benefit (SCB) on the Clinical Global Impressions scales.

**Results:**

Obesity was a significant predictor of reduced preoperative-to-postoperative improvement in the PROMIS Upper Extremity score (*P* = .04). However, BMI was not predictive of other preoperative-to-postoperative differences in outcome scores or the size and number of cuff tendons torn (*P* > .05). Optimal BMI cutoffs were determined for pain MCID (40.8), pain SCB (26.8), function MCID (27.4), and function SCB (26.8), but all cutoffs had low correct classification rates (≤13%).

**Discussion and Conclusion:**

Obesity was not found to be an independent risk factor for increased rotator cuff tear size or tendon involvement but was nonetheless associated with worse upper extremity function and pain after aRCR.

Defined by the World Health Organization as a body mass index (BMI) ≥30, obesity has long posed an arduous challenge to the United States’ healthcare system.^8^ A recent analysis revealed that the current prevalence of obese adults is 35% and that by 2030, this number would increase to 50%.^15^^,^^20^ Literature has shown that obesity can have a devastating impact on the musculoskeletal system as these individuals are predisposed to hip and knee osteoarthritis, degenerative disc disease, lower back pain, and gout.^1^

Rotator cuff tears (RCTs) are a common source of pain and upper extremity disability in obese and nonobese patients alike.^12^^,^^22^ The majority of RCTs are of atraumatic etiology, typically arising from gradual tendon senescence with aging and as such, and full-thickness tears have been reported in 50% of people in their 80s.^9^^,^^14^ Fibroblast apoptosis, decreased cellular activity, collagen disorganization, decreased extracellular matrix synthesis, and the avascularity of the rotator cuff (RC) tendons’ insertion onto the proximal humerus have been reported as plausible etiologies by which this occurs.^17^

Given that the obese state has been shown to promote an inflammatory environment unfavorable to RC tendon healing, there is a concern that obese humans may be at risk of poorer outcomes after undergoing arthroscopic rotator cuff repair (aRCR).^4^ Therefore, the purpose of our study is to examine the effects an increased BMI has on outcomes after aRCR. We hypothesize that an increased BMI will confer higher levels of postoperative pain and lower outcomes scores but that the effect size may vary according to the RCT size and/or type of tendon involved.

## Methods

### Study design and cohort selection

Internal institutional review board approval was granted for this study (institutional review board number: s20-00287). This was a prospective study conducted on a sample of patients that was retrospectively identified utilizing CPT code 29827 between October 2017 and January 2020. Final follow-up data (Patient-Reported Outcomes Measurement Information System [PROMIS] scores and patient satisfaction) were collected prospectively through either phone calls or office visits if they met the following inclusion criteria: (1) age ≥18 years, (2) undergoing primary aRCR surgery with or without long head of biceps tendon (LHBT) or labral debridement and subacromial decompression, (3) minimum 1-year follow-up, (4) had completed the preoperative PROMIS Upper Extremity (UE), Pain Interference, and Pain Intensity surveys, and (5) were able to provide informed consent. Exclusion criteria included (1) patients who underwent open RCR procedures, (2) patients who underwent revision procedures, (3) patients who underwent arthroscopic shoulder procedures that did not repair any RC tendons (eg, subacromial decompression), and (4) patients <18 years of age, and (5) non-English speakers.

### Operative technique

All RCT repairs were performed arthroscopically in the beach chair or lateral decubitus position under regional anesthesia. The kind of repair (single row vs. double row) was determined by the surgeon's preference and the extent of tear/number of tendons involved. The decision for subacromial decompression and biceps tenotomy vs. tenodesis was determined by the operating surgeon. Following surgery, patients’ arms were immobilized for 4-6 weeks (depending on the extent of the tendons involved) and supervised physical therapy was initiated at 4 weeks for small-sized tears and 6 weeks for medium- to large-sized tears.

### Perioperative variables measured

BMI at the time of surgery was abstracted from preoperative assessments from electronic medical records. Subjects were classified into three groups based on BMI: normal (BMI <25), overweight (BMI ≥25 and < 30), and obese (BMI ≥30).^2^ Other demographics (eg, age at the time of surgery, sex) and operative variables (number of tendons repaired, concomitant procedures, and procedure time) were also obtained from electronic medical records. Goutallier stage of RC muscle fatty degeneration and Patte classification of supraspinatus RC retraction were also abstracted from preoperative magnetic resonance imaging reports.

### Outcomes measured

Preoperative imaging (magnetic resonance imaging scans) and intraoperative reports were used to identify the total number of RC tendons torn, specific tendons torn (supraspinatus, infraspinatus, subscapularis, and teres minor), and tears of the LHBT and glenoid labrum. RCTs included full-thickness and partial-thickness tears comprising more than 50% of the tendon footprint but excluded tendinitis or partial tears involving less than 50% of the tendon footprint. Tear sizes were classified as per the DeOrio and Cofield classification of partial, small, medium, large, and massive.

Shoulder pain and functional outcomes were assessed using the PROMIS Pain Interference, Pain Intensity, and UE surveys. These surveys were administered during the preoperative time period and completed online or over the phone after a minimum of 1 year postoperatively. Patients also reported any perceived change in shoulder pain and functional status after surgery using the Clinical Global Impressions (CGI) scale, a 7-point scale with options ranging from “much worse” to “much better.”^5^ CGI scale responses were scored from −3 to 3. A score of 1 (“slightly better”) was deemed to be the minimum clinically important difference (MCID), while a score of 2 (“better”) was considered to be the substantial clinical benefit (SCB).

### Statistical methods

Normality of continuous variables was assessed using the Shapiro-Wilk test. Baseline demographics, operative variables, and preoperative PROMIS scores were compared between the BMI groups using the Kruskal-Wallis test and Dwass-Steel-Critchlow-Fligner post hoc testing. BMI group, age, sex, smoking history (ever-smoker vs. never-smoker), and comorbidities (diabetes, hyperlipidemia, and hypertension) were entered into a multiple linear regression model for continuous outcomes (PROMIS and CGI scores) and a multivariable logistic regression model for binary outcomes (eg, supraspinatus tendon tear). The cutoff BMI values that optimized the number of patients who achieved MCID and SCB for pain and function were determined using receiver operating characteristic (ROC) analysis with Youden’s J-statistic. ROC curves with the area under the curve (AUC) ≥ 0.7 were considered to have an adequate predictive value. For all analyses, *P* values less than 0.05 were considered significant.

## Results

### Cohort characteristics

A total of 313 patients were included in the cohort; of them, 86 (27%) had normal BMI, 127 (41%) were overweight, and 100 (32%) were obese (Table I). The cohort’s mean age was 60 ± 9 years and mean BMI was 29 ± 6. At baseline, there were significant differences between the three groups, with the obese cohort having a significantly higher overall rate of comorbidities (*P* = .001) and a younger mean age than patients with lower BMI (*P* = .03; Table I).

**Table I:** Baseline demographics.

Variables examined	All (n = 313)	BMI <25 (n = 86)	BMI 25-30 (n = 127)	BMI ≥30 (n = 100)	*P* value
BMI	29 ± 6	23 ± 2	28 ± 1	36 ± 4	n/a
Age at the time of surgery	60 ± 9	61 ± 9	60 ± 9	58 ± 9	.04
Sex					.007
Male	185 (59%)	42 (49%)	88 (69%)	55 (55%)	
Female	128 (41%)	44 (51%)	39 (31%)	45 (45%)	
Race/ethnicity					.26
White	227 (73%)	63 (73%)	98 (77%)	66 (66%)	
African-American	31 (10%)	6 (7%)	8 (6%)	17 (17%)	
Asian or Pacific Islander	15 (5%)	5 (6%)	7 (6%)	3 (3%)	
Other	31 (10%)	10 (12%)	10 (8%)	11 (11%)	
Not reported	9 (3%)	2 (2%)	4 (3%)	3 (3%)	
Smoking history					.55
Never	187 (60%)	48 (57%)	73 (57%)	66 (67%)	
Former	98 (32%)	29 (34%)	43 (34%)	26 (27%)	
Current	25 (8%)	8 (9%)	11 (9%)	6 (6%)	
Any comorbidity	179 (57%)	38 (44%)	72 (57%)	69 (69%)	.003
Diabetes mellitus	45 (14%)	12 (14%)	12 (9%)	21 (21%)	.05
Hypertension	124 (40%)	19 (22%)	48 (38%)	57 (57%)	<.001
Hyperlipidemia	125 (40%)	28 (33%)	53 (42%)	44 (44%)	.25
Goutallier stage					.17
0	120 (65%)	37 (73%)	52 (67%)	31 (54%)	
1	34 (18%)	10 (20%)	14 (18%)	10 (18%)	
2	28 (15%)	4 (8%)	11 (14%)	13 (23%)	
3	4 (2%)	0 (0%)	1 (1%)	3 (5%)	
Patte classification					.09
0	111 (49%)	34 (51%)	35 (37%)	42 (63%)	
1	43 (19%)	11 (16%)	23 (24%)	9 (13%)	
2	50 (22%)	14 (21%)	24 (26%)	12 (18%)	
3	24 (11%)	8 (12%)	12 (13%)	4 (6%)	
PROMIS scores before surgery					
Pain Interference	59 ± 7	59 ± 7	59 ± 7	60 ± 7	.54
Pain Intensity	53 ±	52 ± 7	51 ± 7	54 ± 8	.04
Upper Extremity Function	34 ± 8	35 ± 8	35 ± 9	33 ± 7	.08
Procedure laterality					.02
Left	124 (40%)	23 (27%)	55 (43%)	46 (46%)	
Right	189 (60%)	63 (73%)	72 (57%)	54 (54%)	
Procedure time (min)	79 ± 33	86 ± 40	78 ± 29	74 ± 30	.28
Time from surgery to postoperative survey completion (mo)	28 ± 9	29 ± 9	28 ± 9	27 ± 9	.41

*BMI*, body mass index; *PROMIS*, Patient-Reported Outcomes Measurement Information System.

Data are presented as means ± standard deviation. *P* values for the Kruskal-Wallis test (continuous) or chi-squared test or Fisher’s exact test (categorical).

### Surgical outcomes

RCT patterns were similar between the BMI groups (Table II). The majority of patients experienced a tear of a single RC tendon (177 patients; 57%), and almost all patients had a supraspinatus tendon tear (305 patients; 97%). Concomitant LHB pathology was present in 190 patients (61%), and labral pathology was seen in 131 patients (42%) of the cohort. Without adjusting for confounders, there were no significant differences in the total number of cuff tendons torn or tears in individual cuff tendons, LHBT, or labrum between the three BMI groups (*P* > .05). Multivariable logistic regression analysis (Table III) found that BMI was not an independent predictor for the odds of tearing more than 1 cuff tendon, infraspinatus tendon tear, subscapularis tendon tear, LHBT tear, labral tear, or large or massive tear (*P* > .05).

**Table II:** Intraoperative findings and outcomes.

Variables examined	Normal BMI <25 (n = 86)	Overweight BMI 25-30 (n = 127)	Obese BMI ≥30 (n = 100)		*P* value
			BMI 30-40 (n = 85)	BMI ≥40 (n = 15)	
# of cuff tendons torn					.86
1	48 (56%)	71 (56%)	53 (62%)	5 (33%)	
2	30 (35%)	41 (32%)	26 (31%)	7 (47%)	
3	7 (8%)	15 (12%)	6 (7%)	2 (13%)	
4	1 (1%)	0 (0%)	0 (0%)	1 (7%)	
Structures torn					
Supraspinatus	85 (99%)	122 (96%)	83 (98%)	15 (100%)	.49
Infraspinatus	30 (35%)	42 (33%)	25 (29%)	9 (60%)	.96
Subscapularis	17 (20%)	32 (25%)	14 (16%)	4 (27%)	.38
Teres minor	1 (1%)	1 (1%)	1 (1%)	1 (7%)	.82
Biceps tendon	55 (64%)	74 (58%)	51 (60%)	10 (67%)	.70
Labrum	37 (43%)	51 (40%)	38 (45%)	5 (33%)	.88
Tear size					.37
Partia	14 (17%)	26 (21%)	24 (29%)	5 (38%)	
Small	13 (16%)	14 (12%)	6 (7%)	0 (0%)	
Medium	32 (39%)	43 (36%)	32 (39%)	4 (31%)	
Large	9 (11%)	18 (15%)	11 (13%)	0 (0%)	
Massive	14 (17%)	20 (17%)	9 (11%)	4 (31%)	
PROMIS scores after surgery					
Pain Interference	43 ± 8	45 ± 8	47 ± 9	52 ± 11	.01
Pain Intensity	34 ± 6	35 ± 7	37 ± 8	42 ± 10	.03
Upper Extremity Function	51 ± 10	49 ± 10	47 ± 11	41 ± 13	.006
PROMIS score preoperative-to-postoperative difference					
Pain Interference	−15 ± 8	−14 ± 9	−13 ± 10	−9 ± 13	.12
Pain Intensity	−18 ± 8	−16 ± 9	−17 ± 8	−16 ± 11	.34
Upper Extremity Function	16 ± 11	14 ± 10	14 ± 12	10 ± 13	.12
CGI scale for pain	2.7 ± 0.9	2.6 ± 0.9	2.6 ± 0.8	1.7 ± 1.7	.01
CGI scale for function	2.6 ± 0.9	2.4 ± 1.1	2.5 ± 1.0	1.5 ± 1.8	.08

*BMI*, body mass index; *CGI*, Clinical Global Impressions; *PROMIS*, Patient-Reported Outcomes Measurement Information System.

Data are presented as means ± standard deviation. P values for the Kruskal-Wallis test (continuous) or chi-squared test or Fisher’s exact test (categorical).

**Table III:** Multivariable regression results for the BMI group as a predictor of postoperative outcomes.

Outcome	Odds ratio or β coefficient with 95% CI and *P* value
≥1 cuff tendon torn	Obese vs. normal: OR = 0.9 [0.5 to 1.7], *P* = .95 Overweight vs. normal: OR = 1.0 [0.6 to 1.8], *P* = .79
Structures torn	
Infraspinatus	Obese vs. normal: OR = 1.1 [0.6 to 2.2], *P* = .69 Overweight vs. normal: OR = 0.9 [0.5 to 1.8], *P* = .86
Subscapularis	Obese vs. normal: OR = 0.7 [0.3 to 1.6], *P* = .39 Overweight vs. normal: OR = 1.2 [0.6 to 2.4], *P* = .58
Biceps tendon	Obese vs. normal: OR = 1.0 [0.5 to 2.0], *P* = .93 Overweight vs. normal: OR = 0.8 [0.4 to 1.5], *P* = .47
Labrum	Obese vs. normal: OR = 0.9 [0.5 to 1.8], *P* = .85 Overweight vs. normal: OR = 0.9 [0.5 to 1.6], *P* = .68
Large or massive tear size	Obese vs. normal: OR = 0.5 [0.2 to 1.0], *P* = .06 Overweight vs. normal: OR = 0.7 [0.3 to 1.5], *P* = .34
PROMIS scores after surgery	
Pain Interference	Obese vs. normal: β = 3.4 [0.8 to 6.0], *P* = .01 Overweight vs. normal: β = 2.4 [0.0 to 4.8], *P* = .05
Pain Intensity	Obese vs. normal: β = 3.0 [0.6 to 5.4], *P* = .01 Overweight vs. normal: β = 1.5 [−0.7 to 3.7], *P* = .15
Upper Extremity Function	Obese vs. normal: β = −5.2 [−8.4 to -2.0], *P* < .001 Overweight vs. normal: β = −3.0 [−6.0 to 0.0], *P* = .05
PROMIS score preoperative-to-postoperative difference	
Pain Interference	Obese vs. normal: β = 2.4 [−0.6 to 5.4], *P* = .11 Overweight vs. normal: β = 2.2 [−0.4 to 4.8], *P* = .10
Pain Intensity	Obese vs. normal: β = 1.3 [−1.5 to 4.1], *P* = .37 Overweight vs. normal: β = 2.1 [−0.5 to 4.7], *P* = .10
Upper Extremity Function	Obese vs. normal: β = −3.4 [−6.6 to -0.2], *P* = .04 Overweight vs. normal: β = −2.7 [−5.7 to 0.3], *P* = .07
CGI scale for pain	Obese vs. normal: β = −0.2 [−0.4 to 0.0], *P* = .16 Overweight vs. normal: β = −0.1 [−0.3 to 0.1], *P* = .34
CGI scale for function	Obese vs. normal: β = −0.3 [−0.7 to 0.1], *P* = .11 Overweight vs. normal: β = −0.3 [−0.7 to 0.1], *P* = .11

*BMI*, body mass index; *CGI*, Clinical Global Impressions; *PROMIS*, Patient-Reported Outcomes Measurement Information System.

Logistic regression results are reported with an odds ratio (OR) and 95% confidence interval (CI). Linear regression results are reported with a regression coefficient (β) with 95% CI.

### Patient-reported outcomes

There were notable differences in postoperative PROMIS and CGI scores between the BMI groups (Table II). Without adjusting for confounders, postoperative PROMIS Pain Interference and UE scores were lower in the obese group than in the nonobese group (*P* < .05), but these differences were lower than the MCID values for individual outcomes. In addition, the CGI scale for function showed lower perceived improvement in postoperative function among patients in the obese group vs. patients with normal BMI (*P* = .04).

Multiple linear regression analysis (Table III) found obesity to be a significant independent predictor of poorer postoperative PROMIS scores than those of with patients with normal BMI (*P* < .05). Furthermore, obesity was predictive of reduced preoperative-to-postoperative improvement in the PROMIS UE score (β = −3.6, 95% confidence interval [−6.8 to −0.4], *P* = .03) compared with patients with normal BMI. However, BMI was not predictive of other preoperative-to-postoperative differences in PROMIS scores or of CGI scores for pain and functional improvement (*P* > .05).

### BMI cutoff and MCID/SCB

**Figure 1:**
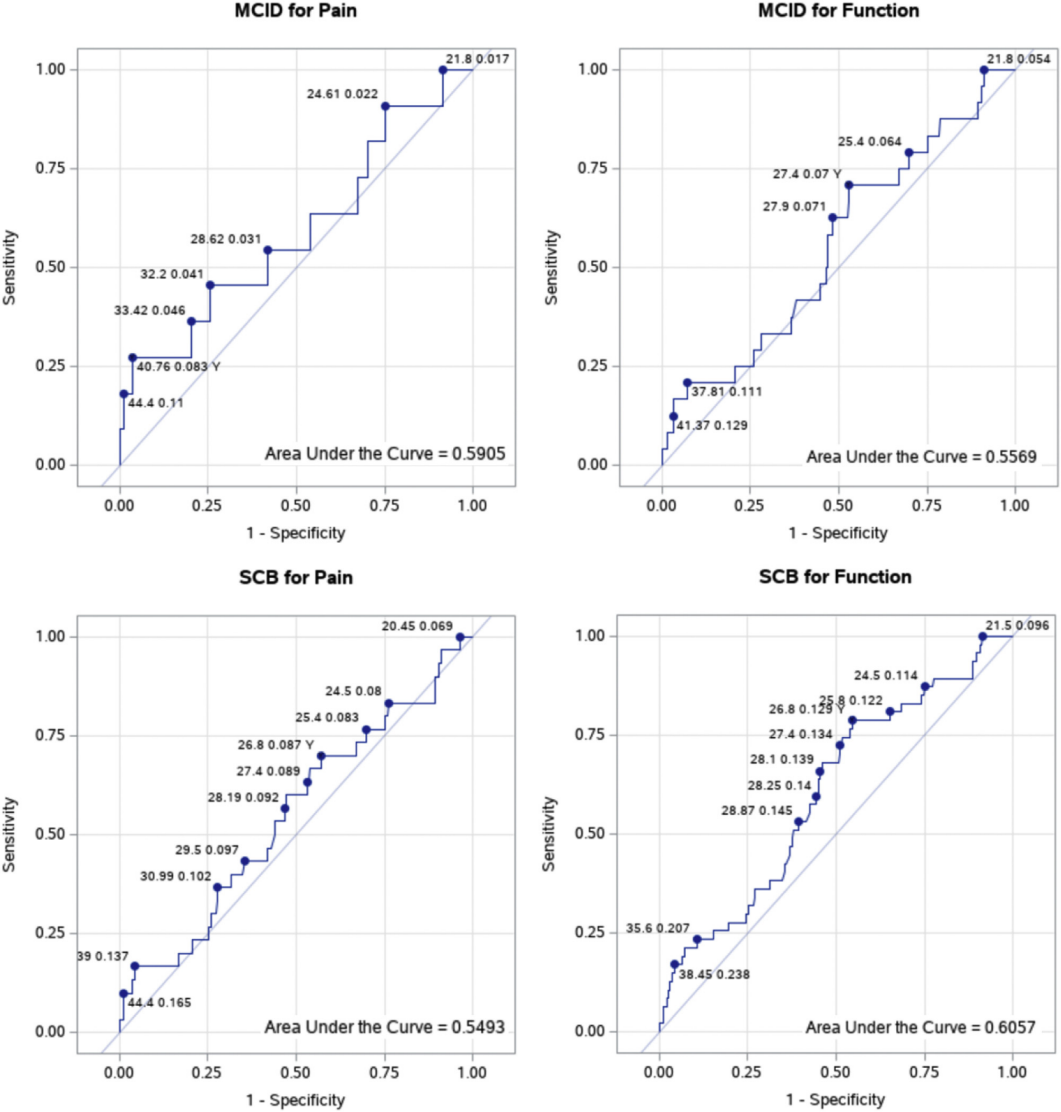
Receiver operating characteristic (*ROC*) curves for BMI as a predictor for achieving the minimum clinically important difference (*MCID*) and significant clinical benefit (*SCB*) for pain and function as measured by the Clinical Global Impression (*CGI*) scale. The area under the curve (*AUC*) is reported for each ROC curve. BMI cut points are marked on each curve along with the correct classification rate associated with the cut point. *BMI*, body mass index.

ROC curves were generated for BMI as a predictor of patients achieving MCID and SCB for pain and function after aRCR (Fig. 1). All models had an AUC <0.7. Based on Youden’s J-statistic, the optimal BMI cut points were determined for pain MCID (BMI 40.8, J = 0.24, correct classification rate = 8.3%), pain SCB (BMI 26.8, J = 0.13, correct classification rate = 8.7%), function MCID (BMI 27.4, J = 0.18, correct classification rate = 7.0%), and function SCB (BMI 26.8, J = 0.24, correct classification rate = 12.9%).

## Discussion

As obesity rates are projected to continue to climb throughout the next decade, it is imperative that orthopedic surgeons understand its ramifications on aRCR outcomes.^15^ The findings of this study indicate that obesity (BMI>30) was not an independent risk factor for an increased severity of RCT size or a higher number of tendon involvement. However, a BMI >30 was associated with lower functional outcome scores and higher pain levels regardless of tear size or presence of concomitant pathology. Obesity was also associated with a poorer patient-perceived clinical improvement.

ROC analysis identified an obese BMI cutoff (40.8) for patients who achieved MCID for pain. However, the same analysis identified overweight BMI cutoffs (<30) for patients who achieved SCB for pain and MCID and SCB for function. This analysis reinforces the existing notion that obese patients experience more pain and have suboptimal functional outcomes after aRCR. It must be stated that these results should be interpreted with caution as the models used in our analysis had poor discrimination and did not attain an ideal predictive value based on the AUC (range: 0.55-0.60). Furthermore, each identified BMI cutoff had a low correct classification rate, with the highest being 12.9% for function SCB. A similar conclusion was reached by Rubenstein et al,^18^ who conducted a retrospective analysis to identify BMI cutoffs for shoulder arthroscopy that would reduce postoperative complications. The authors found a significant cutoff at a BMI of 40 but noted this cutoff had a low positive predictive value (2.3%) and would avoid 12% of major complications while excluding 8% of complication-free surgeries, thus making it unsuitable for preoperative screening. Although there are limitations for application of cutoff values for preoperative planning, this should not underscore the importance of using this information to counsel patients on the effects obesity can have on postoperative outcomes.

As it stands, there are conflicting results in literature regarding the impact of obesity on outcomes after aRCR. A retrospective study of 149 patients by Warrender et al^21^ reported lower functional outcome scores in obese patients than their nonobese counterparts as per mean American Shoulder and Elbow Surgeons scores of 81 ± 27 vs. 90 ± 22 (*P* = .019), respectively. They also found that surgeries in obese patients were longer and required lengthier hospital stays. A study by Ateschrang et al^2^ comparing functional outcomes in patients who underwent open and arthroscopic RCR showed that obese patients, regardless of modality, experienced lower functional outcomes than their nonobese counterparts. Berglund et al^3^ also investigated the effects of various comorbidities on 1-year functional outcomes following aRCR. Here, the authors reported that their obese subset had significantly lower American Shoulder and Elbow Surgeons function scores (39.4 vs. 41.9) (*P* < .05). However, these results serve as a contrast to the findings of the studies by Kessler et al^11^ and Namdari et al^13^ which both reported no differences in patient-reported outcome measures at the latest follow-up between obese and nonobese patients. We hypothesized that these conflicting results could be related to the effect size that obesity may have. It is possible that in small- and medium-sized tears, the impact of obesity may be less pronounced to be clinically significant. In contrast to our hypothesis, we found that irrespective of tear size, obesity was associated with significantly poor outcomes of the PROMIS UE score and higher pain levels, but the effect size did not reach clinically meaningful values (MCID).

Similar to prior works, our overall readmission and complication rate was low with no differences between cohorts (*P* > .05).^11^^,^^21^ However, a recent retrospective study by Kashanchi et al utilizing a large national database demonstrated that obese and morbidly obese patients are at an increased risk of medical, renal, and pulmonary complications, in addition to nonhome discharges.^10^ We feel that the dissonance between our results and theirs is likely attributable to the large sample size (18,521 patients) analyzed in their study.^10^

Another important aspect of this study, in contrast to the work of Gumina et al,^7^ is that an elevated BMI did not confer a greater severity of tear size nor a greater number of tendon involvement or a particular tendon involvement (subscapularis vs. supraspinatus vs. infraspinatus). This is of particular concern as the obese cohort still reported a greater degree of postoperative pain and disability despite their tear sizes and Goutallier/Patte stages featuring no significant differences between nonobese patients. This, in itself, may be due to the inherent molecular changes associated with the obese state. While inflammatory changes have commonly been cited as a main driver for pain and disability, central fatigue at the neuromuscular junction has also been postulated to contribute to shoulder disability in obese patients.^16^ Though the exact etiology of obesity-related disability and pain has yet to be revealed, it is imperative that these patients be monitored closely and receive intensive postoperative rehabilitation to attain optimal outcomes. In fact, a randomized control study by Sweeney et al^19^ lends evidence to the necessity of diligent and effective physical therapy in obese patients. When aggressive resistance and aerobic exercises were implemented in female patients with upper extremity disability following breast cancer surgeries, obese and overweight patients reported lower Disabilities of the Arm, Shoulder and Hand scores and increased range of motion when compared with similar cohorts who did not participate in this program.^19^ As such, more work is needed to fully elucidate the significance of obesity’s effect on disability and pain in the context of aRCR.

We acknowledge several limitations of our study. First, patient reported outcome measures are subject to variability by recent events and influenced by longitudinal changes in shoulder function over time.^6^ To mitigate this, we attempted to account for the effects of possible confounding variables by using multivariable regression techniques in our analysis. Second, we did not have a subgroup of morbid obesity given how few patients fell into this category. Third, we did not account for the chronicity of the RC injury and concomitant pathology. Fourth, we could not control for variance in surgeon preference for biceps (tenotomy vs. tenodesis – arthroscopic or open), labrum tears and subacromial decompression, all of which may affect PROMs. Finally, although the large majority of aRCR procedures performed at our institution involved chronic injuries, it is likely that patients with acute injuries were also captured in our study. While this may affect the homogeneity of our sample, it provides us with a diverse sample reflective of all patients seen in clinical practice.

## Conclusion

Obesity was not found to be an independent risk factor for increased RCT size or tendon involvement but was nonetheless associated with worse upper extremity function and pain after aRCR.

## Disclaimers:

Funding: No funding was disclosed by the authors.

Conflicts of interest: The authors, their immediate families, and any research foundation with which they are affiliated have not received any financial payments or other benefits from any commercial entity related to the subject of this article.
